# Li_0.5_Al_0.5_Mg_2_(MoO_4_)_3_

**DOI:** 10.1107/S1600536813022046

**Published:** 2013-08-14

**Authors:** Ines Ennajeh, Mohamed Faouzi Zid, Ahmed Driss

**Affiliations:** a Laboratory of Materials and Crystallochemistry, Faculty of Science of Tunis, University of Tunis ElManar, 2092 ElManar II Tunis, Tunisia

## Abstract

The title compound, lithium/aluminium dimagnesium tetra­kis­[orthomolybdate(VI)], was prepared by a solid-state reaction route. The crystal structure is built up from MgO_6_ octa­hedra and MoO_4_ tetra­hedra sharing corners and edges, forming two types of chains running along [100]. These chains are linked into layers parallel to (010) and finally linked by MoO_4_ tetra­hedra into a three-dimensional framework structure with channels parallel to [001] in which lithium and aluminium cations equally occupy the same position within a distorted trigonal–bipyramidal coordination environment. The title structure is isotypic with LiMgIn(MoO_4_)_3_, with the In site becoming an Mg site and the fully occupied Li site a statistically occupied Li/Al site in the title structure.

## Related literature

For complex oxides containing lithium ions, see: Whittingham & Silbernagel (1976 ▶); Mizushima *et al.* (1980 ▶); Kanno *et al.* (1994 ▶). For details of chemically and/or structurally related compounds, see: Efremov & Trunov (1972 ▶); Ozima & Zoltai (1976 ▶); Klevtsov (1970 ▶); Kolitsch & Tillmanns (2003 ▶); Tsyren­ova *et al.* (2001 ▶, 2004 ▶); Gicquel-Mayer *et al.* (1981 ▶); Klevtsova & Magarill (1970 ▶); Klevtsov & Zolotova (1973 ▶); Klevtsova *et al.* (1979 ▶); Nord & Kierkegaard (1984 ▶); Solodov­nikov *et al.* (1997 ▶). For the isotypic structure of LiMgIn(MoO_4_)_3_, see: Khazheeva *et al.* (1985 ▶).

## Experimental

### 

#### Crystal data

Li_0.5_Al_0.5_Mg_2_(MoO_4_)_3_
*M* *_r_* = 545.40
Triclinic, 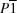

*a* = 6.8555 (7) Å
*b* = 8.2910 (9) Å
*c* = 9.5760 (9) Å
α = 96.032 (7)°
β = 106.743 (8)°
γ = 101.824 (9)°
*V* = 502.27 (9) Å^3^
*Z* = 2
Mo *K*α radiation
μ = 3.92 mm^−1^
*T* = 298 K
0.20 × 0.18 × 0.11 mm

#### Data collection

Enraf–Nonius CAD-4 diffractometer
Absorption correction: ψ scan (North *et al.*, 1968 ▶) *T*_min_ = 0.520, *T*_max_ = 0.648
3450 measured reflections
2187 independent reflections
2150 reflections with *I* > 2sσ(*I*)
*R*_int_ = 0.014
2 standard reflections every 120 min intensity decay: 1.1%

#### Refinement

*R*[*F* ^2^ > 2σ(*F* ^2^)] = 0.021
*wR*(*F* ^2^) = 0.053
*S* = 1.29
2187 reflections
164 parameters
Δρ_max_ = 0.57 e Å^−3^
Δρ_min_ = −0.81 e Å^−3^

### 

Data collection: *CAD-4 EXPRESS* (Duisenberg, 1992 ▶; Macíček & Yordanov, 1992 ▶); cell refinement: *CAD-4 EXPRESS*; data reduction: *XCAD4* (Harms & Wocadlo, 1995 ▶); program(s) used to solve structure: *SHELXS97* (Sheldrick, 2008 ▶); program(s) used to refine structure: *SHELXL97* (Sheldrick, 2008 ▶); molecular graphics: *DIAMOND* (Brandenburg, 1998 ▶); software used to prepare material for publication: *WinGX* (Farrugia, 2012 ▶).

## Supplementary Material

Crystal structure: contains datablock(s) I. DOI: 10.1107/S1600536813022046/wm2760sup1.cif

Structure factors: contains datablock(s) I. DOI: 10.1107/S1600536813022046/wm2760Isup2.hkl

Additional supplementary materials:  crystallographic information; 3D view; checkCIF report

## Figures and Tables

**Table 1:** Selected bond lengths (Å)

Mo1—O5^i^	1.721 (3)
Mo1—O12	1.745 (3)
Mo1—O1	1.781 (3)
Mo1—O2	1.812 (3)
Mo2—O3	1.738 (3)
Mo2—O6	1.743 (3)
Mo2—O11^ii^	1.763 (3)
Mo2—O10^iii^	1.807 (3)
Mo3—O9	1.718 (3)
Mo3—O4	1.736 (3)
Mo3—O7	1.777 (3)
Mo3—O8	1.812 (3)
Mg1—O2^iv^	1.968 (3)
Mg1—O11	1.974 (3)
Mg1—O8^v^	1.983 (3)
Mg1—O4	1.992 (3)
Mg1—O10^iii^	2.033 (3)
Mg1—O2^v^	2.104 (3)
Mg2—O12^vi^	2.042 (3)
Mg2—O1^vii^	2.045 (3)
Mg2—O9	2.046 (3)
Mg2—O5	2.049 (3)
Mg2—O6^viii^	2.049 (3)
Mg2—O7^vi^	2.121 (3)
Li1—O3^ix^	1.974 (4)
Li1—O7^ix^	2.009 (4)
Li1—O1	2.044 (4)
Li1—O8	2.070 (4)
Li1—O10^vi^	2.076 (4)

Symmetry codes: (i) 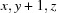; (ii) 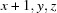; (iii) 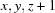; (iv) 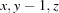; (v) 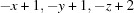; (vi) 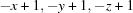; (vii) 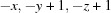; (viii) 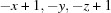; (ix) 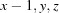.

**Fig. 1.**
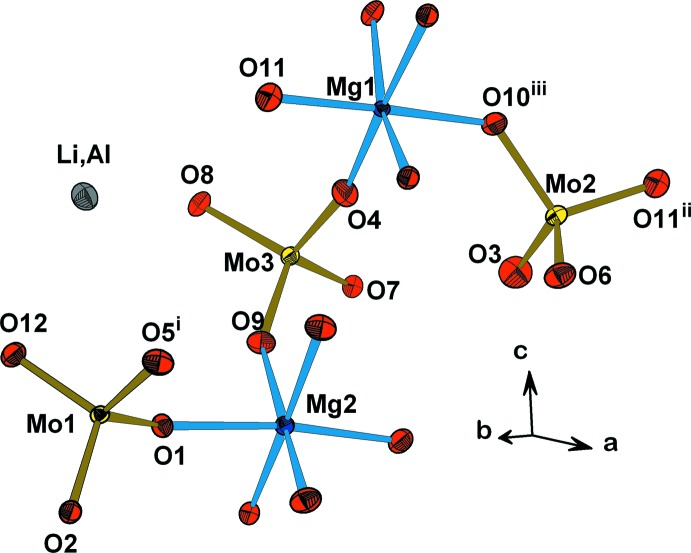
Expanded asymmetric unit of Li0.5Al0.5Mg2(MoO4)3 showing the main building units. All atoms are represented as displacement ellipsoids at the 50% probability level. [Symmetry codes: (i) x, y+1, z; (ii) x+1, y, z; (iii) x, y, z+1.]

**Fig. 2.**
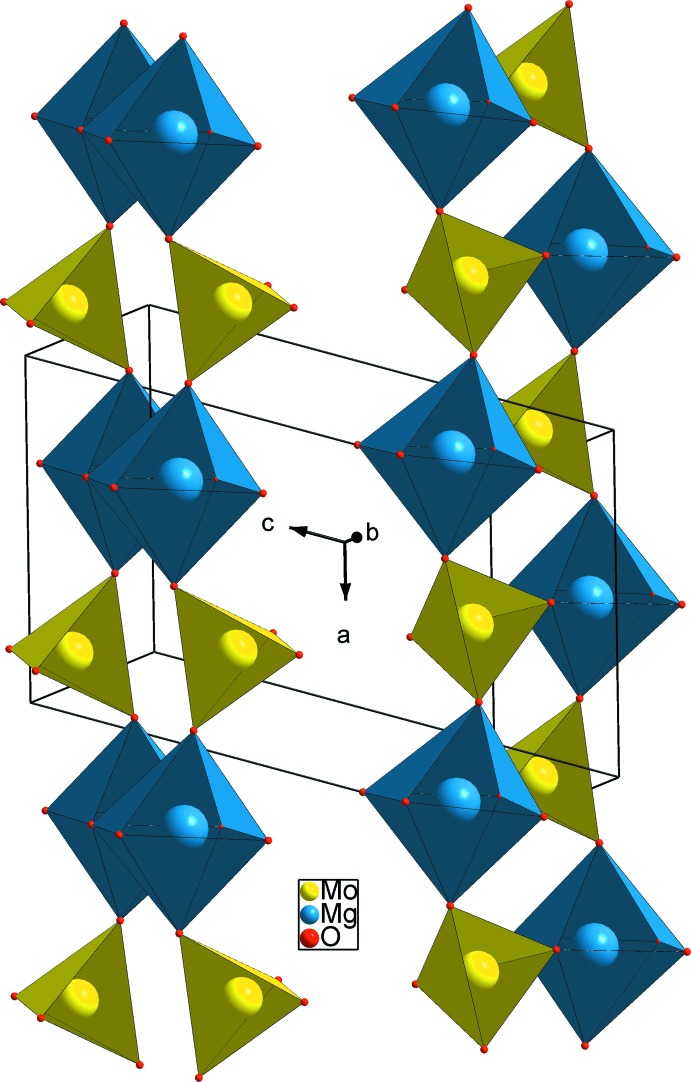
The two types of double chains with composition (Mg2Mo2O14)n, (a) in cis arrangement, (b) in trans arrangement.

**Fig. 3.**
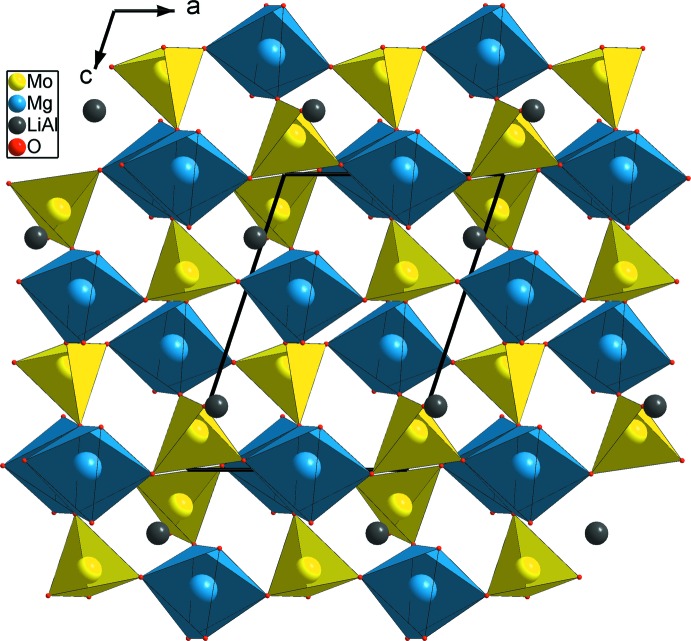
The projection of the Li0.5Al0.5Mg2(MoO4)3 structure showing the layer parallel to (010).

**Fig. 4.**
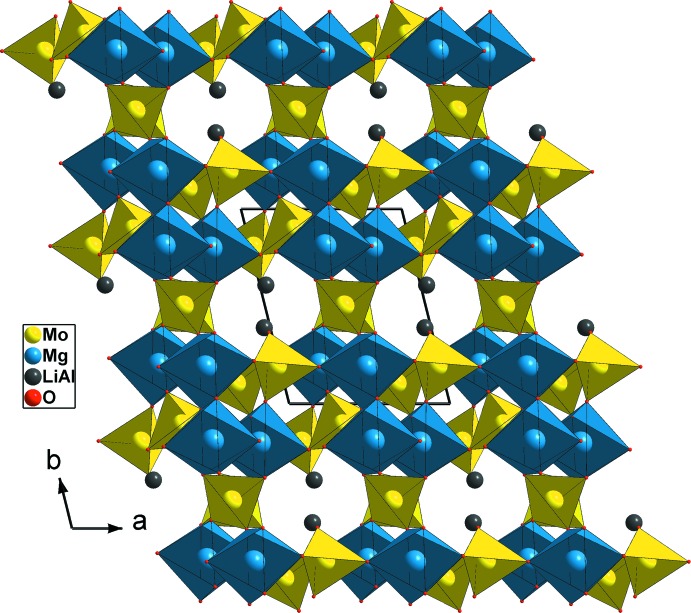
Projection of Li0.5Al0.5Mg2(MoO4)3 along [001].

**Fig. 5.**
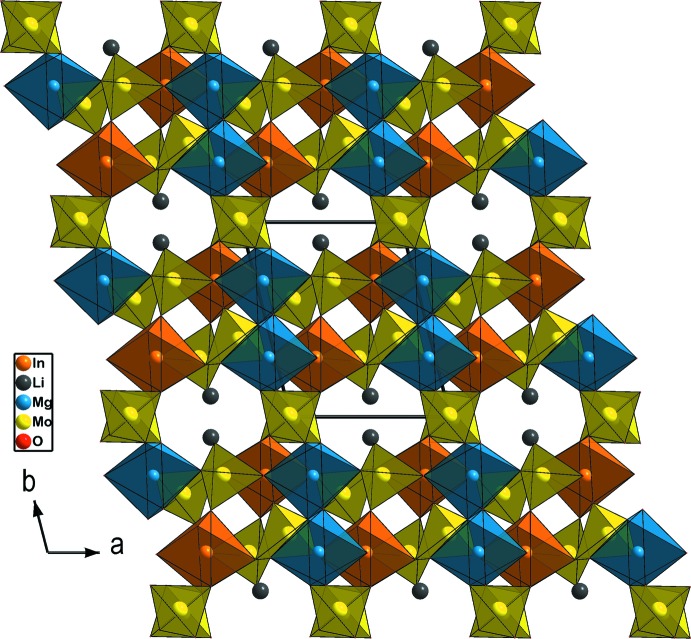
Projection of the isotypic structure of LiMgIn(MoO4)3 along [010].

**Fig. 6.**
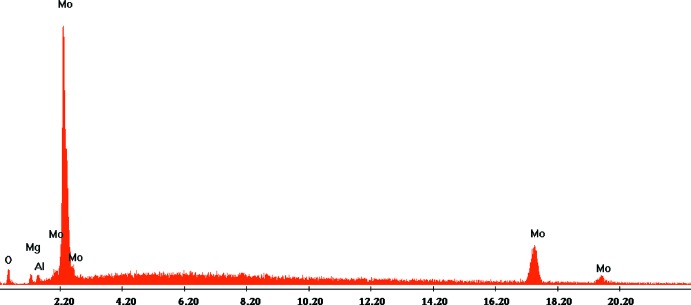
Qualitative EDX analysis of Li0.5Al0.5Mg2(MoO4)3, showing the presence of Al.

## supplementary crystallographic information

### 1. Comment

In recent years, great attention has been devoted to the examination of metal oxides containing mobile lithium ions due to their potential application in the fields of energy and electronics, such as Li*M*O_2_ materials (*M*= Mn, Fe, Co, Ni) (Whittingham & Silbernagel, 1976; Mizushima *et al.*, 1980; Kanno *et al.*, 1994) which allowed to construct electrochemical generators with a high energy density. In our field of research we are interested especially to doubles molybdates of alkali and divalent metals, and we tried to explore systems like Li_2_O—*M*O—MoO_3_. For such systems, the compounds Li_3_Fe(MoO_4_)_3_ and Li_2_Fe_2_(MoO_4_)_3_ are already known, the crystal structures of which have been determined by Klevtsova & Magarill (1970) and the structural similarity to NaCo_2.31_(MoO_4_)_3_ and to other framework oxides is noted. These compounds include Li_2_*M*_2_(MoO_4_)_3_ (*M* = Mg, Mn, Co, Ni, Cu, Zn) (Efremov & Trunov, 1972; Ozima & Zoltai, 1976), Li_3_*M*^3+^(MoO_4_)_3_ (*M*=Al, Cr, Ga, Sc, In, Co) (Klevtsov, 1970; Kolitsch & Tillmanns, 2003) and Li_2_*M*^4+^(MoO_4_)_3_ (*M* = Ti, Zr, Hf) (Klevtsov & Zolotova, 1973; Klevtsova *et al.*, 1979). During our examinations we now have serendipitously obtained a new molybdenum oxide crystal with composition Li_0.5_Al_0.5_Mg_2_(MoO_4_)_3_, (I).

The asymmetric unit of compound (I) is composed of two MgO_6_ octahedra and three MoO_4_ tetrahedra sharing corners, as well as a (li/Al) site (Fig. 1). The structure of (I) can be described as being composed of two types of infinite chains expanding parallel to [100]. The first chain is built up from Mg2O_6_ octahedra and Mo1O_4_ tetrahedra sharing corners, forming double chains with composition (Mg_2_Mo_2_O_14_)_n_ and with a *cis* arrangement of the MoO_4_ tetrahedra relative to the MgO_6_ octahedra (Fig. 2a). The second type of chain is formed by Mg1O_6_ octahedra and Mo2O_4_ tetrahedra, also linked by corners but in a *trans* arrangement. Single chains of the second type are linked by sharing edges between two adjacent Mg1O_6_ octahedra (Fig. 2b). The linkage between the two types of chains leads to a layer-like arrangement parallel to (010) (Fig. 3), whereas the linkage into a three-dimensional framework is provided by Mo3O_4_ tetrahedra by sharing corners. In this framework channels are present where the mixed-occupied Li^+^/Al^3+^ sites are located (Fig. 4). The Mg2O_6_ octahedron has an almost regular coordination sphere with five nearly equal Mg—O distances (*d*(Mg—O) = 2.04 Å) with the sixth slightly longer. Each Mg1_2_O_10_ double octahedron is surrounded by ten MoO_4_ tetrahedra forming Mg1_2_Mo_10_O_40_ units. The Mg—O distances vary from 1.968 (3) Å to 2.104 (3) Å which are close to those found for related systems (Nord & Kierkegaard, 1984). The Mo—O distances vary from 1.719 (3) Å to 1.812 (3) Å with the average Mo—O distance of 1.762 Å, closed to literature values (Solodovnikov *et al.*, 1997). The (Li/Al) site has a distorted trigonal-bipyramidal coordination environment.

The structure of compound (I) is isotypic with LiMgIn(MoO_4_)_3_ (Khazheeva *et al.*, 1985). The In^3+^ site becomes Mg2 in the title structure, and the Li site in LiMgIn(MoO_4_)_3_ has full occupation, whereas in the title structure it is a mixed-occupied (Li/Al) site with half-occupancy for each of the metal ions. In fact, charge compensation can only be ensured by insertion of Al^3+^ in the same site as Li^+^ ((Mg^2+^ + In^3+^ + Li^+^) = (2Mg^2+^ + (Al^3+^/Li^+^)) (Fig. 5).

The unit-cell parameters of triclinic Li_0.5_Al_0.5_Mg_2_(MoO_4_)_3_ indicate some resemblance to the structures of Ag_2_*M*_2_(MoO_4_)_3_ (*M* = Zn, Mg, Co) (Tsyrenova *et al.*, 2004, 2001; Gicquel-Mayer *et al.*, 1981), but a close comparison of the structures reveals some differences. The latter have mixed frameworks of MoO_4_ tetrahedra and pairs of *M*O_6_ octahedra sharing common edges, whereas in structure (I) *M*O_6_ octahedra and also Mg_2_O_10_ units surrounded by MoO_4_ tetrahedra are present. A further comparison of the structure of compound (I) with the Li_2_*M*_2_(MoO_4_)_3_ family (*M*=Mg, Mn, Co, Ni, Cu, Zn) (Efremov & Trunov, 1972; Ozima & Zoltai, 1976) reveals that the substitution of some of the lithium ions by aluminium has changed the crystal structure. The latter family adopts the lyonsite structure type, space group *Pnma*, and their general formula can be written as *A*_16_*B*_12_O_48_. Generally, the *A* site is statistically occupied by Li^+^ and a *M*^2+^ ion.

### 2. Experimental

The title compound, Li_0.5_Al_0.5_Mg_2_(MoO_4_)_3_, was obtained serendipitously by a solid state reaction from appropriate quantities of LiNO_3_ (Fluka, 62575), (NH_4_)_2_Mo_4_O_13_ (Fluka, 69858) and Mg(NO_3_)_2_^.^6H_2_O (Fluka, 63079) placed in a porcelain crucible, and slowly annealed in air at 623 K for 12 h, in order to eliminate volatile products. The resulting mixture was then heated to 853 K for 7 days before slowly cooling at 5 K/day to 773 K. Finally, the furnace was cooled at 50 K/day to room temperature. A qualitative EDX analysis of a selected crystal using a FEI Quanta 200 system revealed the presence of Al, Mo, Mg and O (Fig. 6). Aluminium was not present in the employed educts of the reaction mixture, but the incorporation of aluminium from the porcelain crucible is the most likely source of this element.

### 3. Refinement

During the first stages of refinement the site in the channels was first attributed solely to Li. However, the refined composition did not satisfy electrical neutrality. After considering the presence of Al (see 'experimental part') on this site with an occupancy ratio of 1:1 for Li and Al, electrical neutrality was achieved. Both metals were refined with the same coordinates and the same anisotropic displacement parameters.

### Crystal data

**Table:**

Li_0.5_Al_0.5_Mg_2_(MoO_4_)_3_	*Z* = 2
*M**_r_* = 545.40	*F*(000) = 508
Triclinic, *P*1	*D*_x_ = 3.606 Mg m^−^^3^
Hall symbol: -P 1	Mo *K*α radiation, λ = 0.71073 Å
*a* = 6.8555 (7) Å	Cell parameters from 25 reflections
*b* = 8.2910 (9) Å	θ = 10–15°
*c* = 9.5760 (9) Å	µ = 3.92 mm^−^^1^
α = 96.032 (7)°	*T* = 298 K
β = 106.743 (8)°	Prism, colourless
γ = 101.824 (9)°	0.2 × 0.18 × 0.11 mm
*V* = 502.27 (9) Å^3^	

### Data collection

**Table:**

Enraf–Nonius CAD-4 diffractometer	2150 reflections with *I* > 2sσ(*I*)
Radiation source: fine-focus sealed tube	*R*_int_ = 0.014
Graphite monochromator	θ_max_ = 27.0°, θ_min_ = 2.3°
ω/2θ scans	*h* = −8→4
Absorption correction: ψ scan (North *et al.*, 1968)	*k* = −10→10
*T*_min_ = 0.520, *T*_max_ = 0.648	*l* = −12→12
3450 measured reflections	2 standard reflections every 120 min
2187 independent reflections	intensity decay: 1.1%

### Refinement

**Table:**

Refinement on *F*^2^	Primary atom site location: structure-invariant direct methods
Least-squares matrix: full	Secondary atom site location: difference Fourier map
*R*[*F*^2^ > 2σ(*F*^2^)] = 0.021	*w* = 1/[σ^2^(*F*_o_^2^) + (0.0096*P*)^2^ + 2.5823*P*] where *P* = (*F*_o_^2^ + 2*F*_c_^2^)/3
*wR*(*F*^2^) = 0.053	(Δ/σ)_max_ = 0.001
*S* = 1.29	Δρ_max_ = 0.57 e Å^−^^3^
2187 reflections	Δρ_min_ = −0.81 e Å^−^^3^
164 parameters	Extinction correction: *SHELXL97* (Sheldrick, 2008), Fc^*^=kFc[1+0.001xFc^2^λ^3^/sin(2θ)]^-1/4^
0 restraints	Extinction coefficient: 0.0123 (4)

### Special details

**Table:**

Geometry. All e.s.d.'s (except the e.s.d. in the dihedral angle between two l.s. planes) are estimated using the full covariance matrix. The cell e.s.d.'s are taken into account individually in the estimation of e.s.d.'s in distances, angles and torsion angles; correlations between e.s.d.'s in cell parameters are only used when they are defined by crystal symmetry. An approximate (isotropic) treatment of cell e.s.d.'s is used for estimating e.s.d.'s involving l.s. planes.
Refinement. Refinement of *F*^2^ against ALL reflections. The weighted *R*-factor *wR* and goodness of fit *S* are based on *F*^2^, conventional *R*-factors *R* are based on *F*, with *F* set to zero for negative *F*^2^. The threshold expression of *F*^2^ > σ(*F*^2^) is used only for calculating *R*-factors(gt) *etc*. and is not relevant to the choice of reflections for refinement. *R*-factors based on *F*^2^ are statistically about twice as large as those based on *F*, and *R*- factors based on ALL data will be even larger.

### Fractional atomic coordinates and isotropic or equivalent isotropic displacement parameters (Å^2^)

**Table:**

	*x*	*y*	*z*	*U*_iso_*/*U*_eq_	Occ. (<1)
Mo1	0.30272 (5)	0.89586 (4)	0.66046 (3)	0.00921 (10)	
Mo2	0.98153 (5)	0.19503 (4)	0.87863 (4)	0.01148 (10)	
Mo3	0.49457 (5)	0.50622 (4)	0.74760 (3)	0.00964 (10)	
Mg1	0.53871 (19)	0.19031 (15)	0.99366 (13)	0.0071 (2)	
Mg2	0.2478 (2)	0.20149 (16)	0.40750 (14)	0.0096 (2)	
Li1	0.0379 (4)	0.6097 (3)	0.7889 (3)	0.0200 (5)	0.50
Al1	0.0379 (4)	0.6097 (3)	0.7889 (3)	0.0200 (5)	0.50
O1	0.0505 (4)	0.7660 (3)	0.6386 (3)	0.0131 (5)	
O2	0.4454 (4)	0.9765 (4)	0.8546 (3)	0.0147 (6)	
O3	0.9874 (5)	0.3666 (4)	0.7886 (4)	0.0252 (7)	
O4	0.5435 (5)	0.3564 (4)	0.8579 (3)	0.0212 (6)	
O5	0.2586 (5)	0.0618 (4)	0.5726 (3)	0.0197 (6)	
O6	0.8558 (5)	0.0118 (4)	0.7493 (3)	0.0212 (6)	
O7	0.7368 (4)	0.6232 (4)	0.7376 (3)	0.0138 (5)	
O8	0.3563 (4)	0.6386 (4)	0.8234 (3)	0.0151 (6)	
O9	0.3363 (5)	0.4040 (4)	0.5737 (3)	0.0165 (6)	
O10	0.8481 (5)	0.2198 (4)	0.0140 (3)	0.0180 (6)	
O11	0.2432 (5)	0.1911 (4)	0.9700 (4)	0.0226 (7)	
O12	0.4468 (5)	0.7896 (4)	0.5776 (3)	0.0177 (6)	

### Atomic displacement parameters (Å^2^)

**Table:**

	*U*^11^	*U*^22^	*U*^33^	*U*^12^	*U*^13^	*U*^23^
Mo1	0.00817 (16)	0.01051 (16)	0.00822 (16)	0.00090 (12)	0.00288 (12)	0.00056 (11)
Mo2	0.01273 (17)	0.01071 (17)	0.01133 (17)	0.00244 (12)	0.00502 (12)	0.00086 (12)
Mo3	0.00894 (16)	0.01052 (16)	0.00917 (16)	0.00232 (12)	0.00293 (12)	0.00046 (11)
Mg1	0.0077 (6)	0.0069 (6)	0.0065 (5)	0.0020 (4)	0.0020 (4)	0.0006 (4)
Mg2	0.0088 (6)	0.0097 (6)	0.0094 (6)	0.0014 (5)	0.0027 (5)	0.0008 (5)
Li1	0.0172 (10)	0.0215 (11)	0.0203 (11)	0.0038 (9)	0.0046 (9)	0.0060 (9)
Al1	0.0172 (10)	0.0215 (11)	0.0203 (11)	0.0038 (9)	0.0046 (9)	0.0060 (9)
O1	0.0111 (13)	0.0137 (13)	0.0147 (13)	0.0028 (10)	0.0041 (11)	0.0044 (11)
O2	0.0121 (13)	0.0172 (14)	0.0128 (13)	0.0036 (11)	0.0025 (11)	−0.0018 (11)
O3	0.0305 (18)	0.0179 (15)	0.0298 (17)	0.0038 (13)	0.0127 (15)	0.0103 (13)
O4	0.0207 (15)	0.0250 (16)	0.0204 (15)	0.0089 (13)	0.0064 (13)	0.0084 (13)
O5	0.0244 (16)	0.0160 (14)	0.0180 (15)	0.0025 (12)	0.0064 (12)	0.0054 (12)
O6	0.0240 (16)	0.0198 (15)	0.0170 (15)	0.0020 (13)	0.0071 (13)	−0.0033 (12)
O7	0.0124 (13)	0.0158 (13)	0.0140 (13)	0.0050 (11)	0.0047 (11)	0.0024 (11)
O8	0.0136 (13)	0.0153 (14)	0.0175 (14)	0.0030 (11)	0.0081 (11)	−0.0004 (11)
O9	0.0187 (14)	0.0140 (14)	0.0126 (13)	0.0001 (11)	0.0028 (11)	−0.0011 (11)
O10	0.0222 (15)	0.0206 (15)	0.0150 (14)	0.0092 (12)	0.0084 (12)	0.0040 (12)
O11	0.0200 (15)	0.0275 (17)	0.0219 (16)	0.0080 (13)	0.0079 (13)	0.0031 (13)
O12	0.0147 (14)	0.0219 (15)	0.0157 (14)	0.0038 (12)	0.0056 (11)	−0.0016 (12)

### Geometric parameters (Å, º)

**Table:**

Mo1—O5^i^	1.721 (3)	Mg1—O4	1.992 (3)
Mo1—O12	1.745 (3)	Mg1—O10^iii^	2.033 (3)
Mo1—O1	1.781 (3)	Mg1—O2^v^	2.104 (3)
Mo1—O2	1.812 (3)	Mg2—O12^vi^	2.042 (3)
Mo2—O3	1.738 (3)	Mg2—O1^vii^	2.045 (3)
Mo2—O6	1.743 (3)	Mg2—O9	2.046 (3)
Mo2—O11^ii^	1.763 (3)	Mg2—O5	2.049 (3)
Mo2—O10^iii^	1.807 (3)	Mg2—O6^viii^	2.049 (3)
Mo3—O9	1.718 (3)	Mg2—O7^vi^	2.121 (3)
Mo3—O4	1.736 (3)	Li1—O3^ix^	1.974 (4)
Mo3—O7	1.777 (3)	Li1—O7^ix^	2.009 (4)
Mo3—O8	1.812 (3)	Li1—O1	2.044 (4)
Mg1—O2^iv^	1.968 (3)	Li1—O8	2.070 (4)
Mg1—O11	1.974 (3)	Li1—O10^vi^	2.076 (4)
Mg1—O8^v^	1.983 (3)		
			
O5^i^—Mo1—O12	108.83 (15)	O11—Mg1—O2^v^	94.31 (13)
O5^i^—Mo1—O1	106.36 (14)	O8^v^—Mg1—O2^v^	82.89 (12)
O12—Mo1—O1	111.36 (14)	O4—Mg1—O2^v^	176.13 (14)
O5^i^—Mo1—O2	108.44 (14)	O10^iii^—Mg1—O2^v^	91.76 (12)
O12—Mo1—O2	111.03 (13)	O12^vi^—Mg2—O1^vii^	166.46 (14)
O1—Mo1—O2	110.64 (13)	O12^vi^—Mg2—O9	91.79 (13)
O3—Mo2—O6	109.58 (16)	O1^vii^—Mg2—O9	86.88 (13)
O3—Mo2—O11^ii^	108.05 (16)	O12^vi^—Mg2—O5	92.20 (14)
O6—Mo2—O11^ii^	109.67 (15)	O1^vii^—Mg2—O5	101.11 (13)
O3—Mo2—O10^iii^	108.85 (15)	O9—Mg2—O5	85.35 (13)
O6—Mo2—O10^iii^	111.40 (15)	O12^vi^—Mg2—O6^viii^	91.25 (13)
O11^ii^—Mo2—O10^iii^	109.23 (14)	O1^vii^—Mg2—O6^viii^	91.06 (13)
O9—Mo3—O4	107.98 (15)	O9—Mg2—O6^viii^	175.12 (14)
O9—Mo3—O7	109.75 (14)	O5—Mg2—O6^viii^	90.72 (14)
O4—Mo3—O7	109.09 (14)	O12^vi^—Mg2—O7^vi^	85.67 (13)
O9—Mo3—O8	108.76 (14)	O1^vii^—Mg2—O7^vi^	80.80 (12)
O4—Mo3—O8	109.30 (14)	O9—Mg2—O7^vi^	86.40 (12)
O7—Mo3—O8	111.88 (13)	O5—Mg2—O7^vi^	171.41 (13)
O2^iv^—Mg1—O11	90.13 (14)	O6^viii^—Mg2—O7^vi^	97.64 (13)
O2^iv^—Mg1—O8^v^	163.23 (14)	O3^ix^—Li1—O7^ix^	97.36 (16)
O11—Mg1—O8^v^	92.29 (13)	O3^ix^—Li1—O1	137.17 (18)
O2^iv^—Mg1—O4	102.01 (14)	O7^ix^—Li1—O1	83.58 (14)
O11—Mg1—O4	88.75 (14)	O3^ix^—Li1—O8	93.03 (16)
O8^v^—Mg1—O4	94.64 (14)	O7^ix^—Li1—O8	168.56 (17)
O2^iv^—Mg1—O10^iii^	94.68 (13)	O1—Li1—O8	85.55 (14)
O11—Mg1—O10^iii^	172.82 (15)	O3^ix^—Li1—O10^vi^	121.05 (17)
O8^v^—Mg1—O10^iii^	84.64 (13)	O7^ix^—Li1—O10^vi^	97.16 (15)
O4—Mg1—O10^iii^	85.04 (13)	O1—Li1—O10^vi^	101.10 (15)
O2^iv^—Mg1—O2^v^	80.38 (13)	O8—Li1—O10^vi^	81.44 (14)

Symmetry codes: (i) *x*, *y*+1, *z*; (ii) *x*+1, *y*, *z*; (iii) *x*, *y*, *z*+1; (iv) *x*, *y*−1, *z*; (v) −*x*+1, −*y*+1, −*z*+2; (vi) −*x*+1, −*y*+1, −*z*+1; (vii) −*x*, −*y*+1, −*z*+1; (viii) −*x*+1, −*y*, −*z*+1; (ix) *x*−1, *y*, *z*.
